# Salivary gland ablation: introducing an interventional radiology treatment alternative in the management of sialorrhea

**DOI:** 10.1007/s00247-020-04649-6

**Published:** 2020-03-21

**Authors:** Katherine A. Begley, Leah E. Braswell, Garey H. Noritz, James W. Murakami

**Affiliations:** 1 The Ohio State University College of Medicine, Columbus, OH USA; 2 Department of Radiology, Nationwide Children’s Hospital, 700 Children’s Drive, Columbus, OH 43205 USA; 3 Department of Complex Care, Nationwide Children’s Hospital, Columbus, OH USA

**Keywords:** Ablation, Children, Interventional radiology, Salivary glands, Sclerotherapy, Sialorrhea, Ultrasound

## Abstract

**Background:**

Sialorrhea is common in children with neurological disorders and leads to social isolation, aspiration pneumonia and increased caregiver burden. Sialorrhea management includes anticholinergic medications and a variety of surgeries, but these are limited by side effects, recurrence and risks.

**Objective:**

We present our method of salivary gland ablation, an interventional radiology treatment for sialorrhea, and report safety and efficacy data from pediatric patients who underwent salivary gland ablation.

**Materials and methods:**

Salivary gland ablation uses image-guided sotradecol and ethanol dual-drug injection into the salivary glands. Submandibular and sublingual glands are injected percutaneously using ultrasound. Parotid glands are injected retrograde through Stensen ducts using fluoroscopy. We conducted a retrospective review of the medical records of patients who underwent salivary gland ablation at our institution between 2005 and 2019. Pre- and post-procedure Drooling Frequency and Drooling Severity (DFDS) scale scores were compared and caregiver satisfaction was assessed. We devised two cohorts, one to study patient safety and a subcohort to study clinical efficacy using DFDS scores.

**Results:**

One hundred and seventy salivary gland ablation procedures were performed in the 99 patients comprising the safety cohort. Of the procedures, 88.8% resulted in no or minimal complications. Respiratory difficulty, temporary nerve palsy and infection represent the majority of the 11.2% of patients who experienced periprocedural complications. There were no complications resulting in permanent sequelae. Twenty-seven patients met our inclusion criteria for the efficacy subcohort with a mean follow-up time of 5.4 years. DFDS at follow-up decreased from a median score of nine to a seven post-procedure (*P*=0.000018). The proportion of caregivers who were satisfied with the procedure increased as more glands were ablated, which suggests a causal link between the number of glands ablated and the outcome.

**Conclusion:**

Salivary gland ablation is a safe and effective procedure with the potential for permanent decrease in symptoms related to sialorrhea.

## Introduction

Sialorrhea is the unintentional loss of saliva from the mouth, which is normal under the age of 2 years, but is considered abnormal if it persists beyond the age of 4 years [1, 2]. Sialorrhea is common in children with a variety of neurological disorders including cerebral palsy [2, 3]. In most neurological disorders, the cause is oral sensorimotor impairment, which manifests as the lack of oral continence and a normal swallow reflex [4].

Anterior spillage of saliva from the mouth, drooling, can lead to dermatitis, perioral infection, hygiene compromise, the need for frequent clothing changes, and damage to toys and other belongings. This increases the burden of caregivers [5]. Drooling past an age that is perceived to be normal can impair social interaction and emotional development [6]. Posterior spillage of saliva into the airway can lead to recurrent aspiration, pneumonia and chronic inflammatory lung disease [7]. Depending on a child’s posture and ability to swallow or contain saliva using their lips, they may suffer from anterior spillage, posterior spillage or a mixture [8].

Sialorrhea management usually begins conservatively with postural and behavioral modification and commonly includes anticholinergic medications such as glycopyrrolate, scopolamine and atropine [9]. These medications can be effective but have intolerable side effects for many; urinary retention, constipation and blurred vision are among the most severe [10, 11]. When these therapies are ineffective or intolerable, botulinum toxin A (BoNT-A) may be injected into the submandibular and parotid glands to temporarily decrease saliva production. BoNT-A injections are minimally invasive but must be repeated every 4-6 months and typically require general anesthesia [2]. Surgical procedures, such as salivary duct ligation and salivary gland excision, are more invasive treatments for sialorrhea [12, 13]. These procedures vary in efficacy and recovery and carry risks for adverse events, such as aspiration pneumonia, postoperative hemorrhage, facial nerve palsy, and airway swelling and blockage. A detailed review of the surgical techniques to treat sialorrhea is beyond the scope of this article, but a reading of this literature reveals variability in combinations of surgeries as well as metrics of outcome measurement, which makes definitive summary of surgical outcomes and risks difficult to concisely present [13].

Sclerotherapy is a method of inducing inflammation, cell destruction and tissue fibrosis by injecting a caustic agent into a target tissue [14]. This core interventional radiology (IR) technique is broadly used to treat numerous congenital and acquired disorders including vascular malformations, benign cysts and solid organ disease [14–16]. Salivary gland ablation using sclerotherapy successfully shrank salivary glands in a rat model [17]. In addition, salivary gland ablation has been successfully employed in treating ranulas in children [18]. Using these results as a starting point, we developed a clinically feasible salivary gland ablation treatment using sotradecol and ethanol injection to ablate the sublingual, submandibular and parotid glands of patients with sialorrhea. We present our salivary gland ablation technique, safety data in a cohort of 99 patients, and clinical results in a subcohort of 27 patients.

## Materials and methods

### Salivary gland ablation procedure

Informed consent was obtained from each patient’s guardian before the salivary gland ablation procedure. Salivary gland ablation was primarily done on an outpatient basis, though four children with a history of slow recovery after anesthesia were observed overnight in the hospital. Later in our experience, any patient on nighttime breathing support such as CPAP (continuous positive airway pressure) or BiPAP (bilevel positive airway pressure) was admitted overnight for observation following the procedure. This change primarily reflects a hospital-wide policy shift to admit these patients for observation following any anesthetics. All procedures were done under general anesthesia, preferably with nasotracheal intubation to make Stensen duct cannulation technically easier.

All procedures were performed in the IR suite by one of three attending interventional radiologists (Dr. W. E. Shiels II with 25 years of experience, J.W.M. with 20 years of experience and L.E.B. with 8 years of experience). The majority of patients received a single dose of pre-procedure antibiotic, cefazolin, at the discretion of the radiologist. Real-time ultrasound (US) was used to evaluate the salivary glands and guide the procedure. The number and laterality of glands to be ablated during each treatment were determined based upon a pre-procedural consultation between the patient’s guardian and the IR attending. The following clinical and practical factors were considered: family goals and risk tolerance, and anatomical determinants such as jaw size, gland size and Stensen duct accessibility. Glands that were diminutive by US evaluation were not ablated. Children with micrognathia often have diminutive sublingual glands, which were therefore not treated. Parotid glands were not treated in patients if the duct could not be cannulated successfully. In a small minority of the patients, less than 10%, nutrition was exclusively via a percutaneous feeding tube and the airway was secured with a tracheostomy tube. For these patients, bilateral salivary gland ablation was sometimes performed in a single treatment session as peri-treatment swelling would not limit nutritional intake or respiration. If a patient did not have these support tubes, only one side was treated during any one procedure. In general, if a bilateral treatment was performed, one parotid gland was treated along with both submandibular glands and both sublingual glands. If a unilateral treatment was performed, the parotid gland, submandibular gland and sublingual gland on one side were treated.

Three percent sodium tetradecyl sulfate (STS) (Mylan Institutional LLC, Rockford, IL) and 98% dehydrated ethanol (EtOH) (American Regent Inc., Shirley, NY) were used to ablate the glands, and sclerosant volumes were chosen based upon the sonographic appearance of the gland as well as the judgment of the attending radiologist. Care was taken to inject the maximum amount of sclerosant while avoiding extravasation of medication from the gland capsule. Early in our clinical experience with salivary gland ablation, EtOH alone was used. Anecdotally, we found a greater inflammatory effect after sclerotherapy if STS was used in concert with EtOH. Our salivary gland ablation method, therefore, evolved to use un-foamed STS along with EtOH. As with most sclerotherapy, salivary gland ablation can be a staged procedure. Each patient was evaluated clinically during follow-up appointments and salivary gland ablation was repeated after several months if the desired clinical response was not achieved, usually targeting the glands not treated in previous sessions. The clinical decision to do a second procedure was made between the interventional radiologist and the family based upon their subjective assessment of the value of the clinical outcome after the first treatment weighed against the risks and costs of performing additional procedures.

**Fig. 1:**
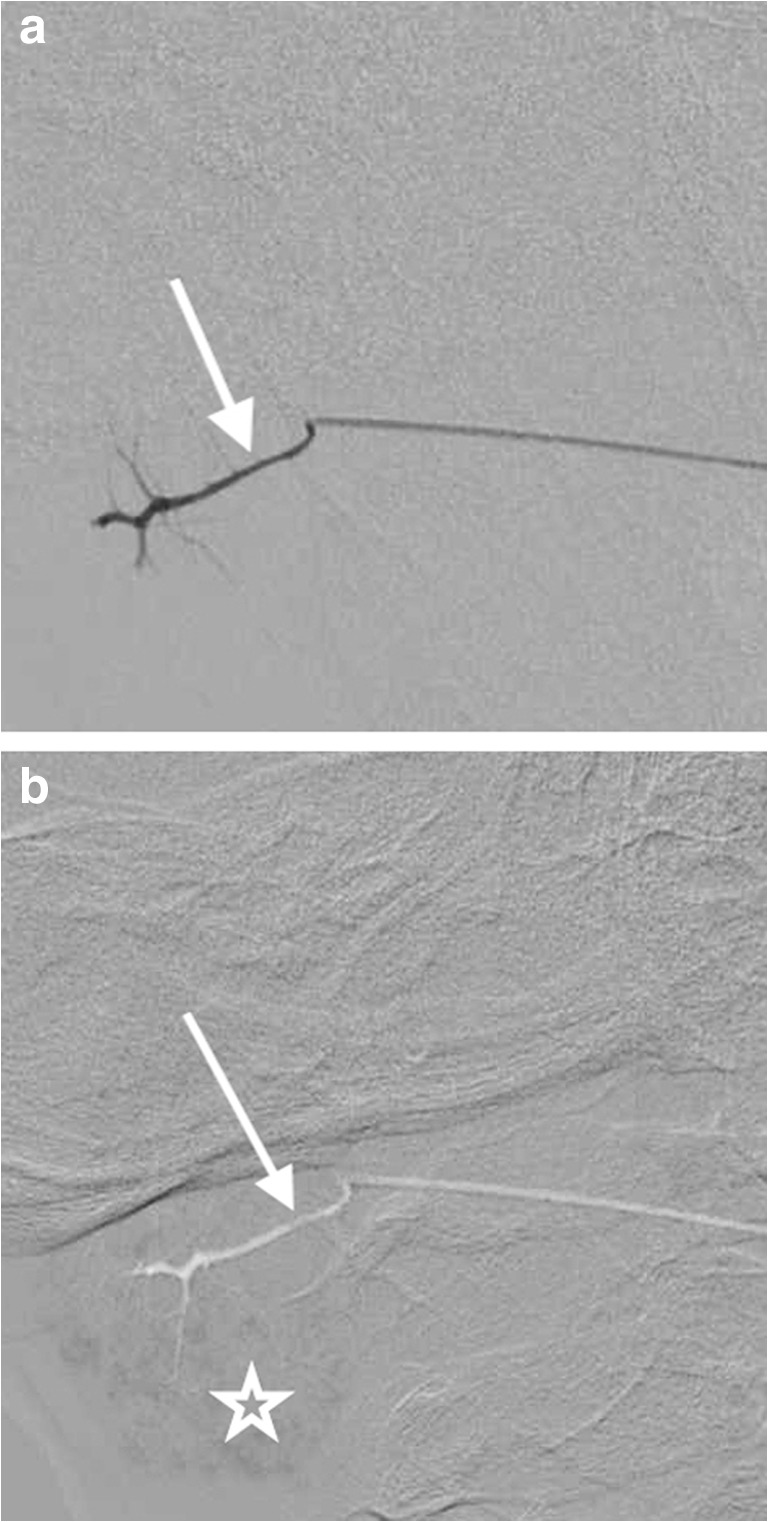
Digital subtraction angiography images during the parotid gland ablation in a 10-year-old boy. **a** Contrast injection through a 3-French dilator opacified Stensen duct (*arrow*) confirms the appropriate dilator position. **b** Injection of unopacified 3% sodium tetradecyl sulfate fills the ductal system (*arrow*) displacing the contrast into the acini (*star*) of the parotid gland

Parotid glands were accessed by advancing an 0.018-in. straight hydrophilic-coated guidewire (Terumo, Somerset, NJ) retrograde into Stensen duct using direct intraoral visual inspection. Wire position was confirmed with fluoroscopy, and then the 3-French (Fr) inner portion of a 4-Fr micropuncture introducer set (Cook, Bloomington, IN) was advanced over the wire. Once in place, a small volume of water-soluble contrast (0.1–0.2 mL) was gently injected by hand into the duct under fluoroscopic guidance to confirm position (Fig. 1). Next, STS was injected into the duct using the digital subtraction angiography (DSA) technique to watch un-opacified STS push the contrast into the gland and opacify the acinar network of the gland (Fig. 1). This process was repeated during EtOH injection.

**Fig. 2:**
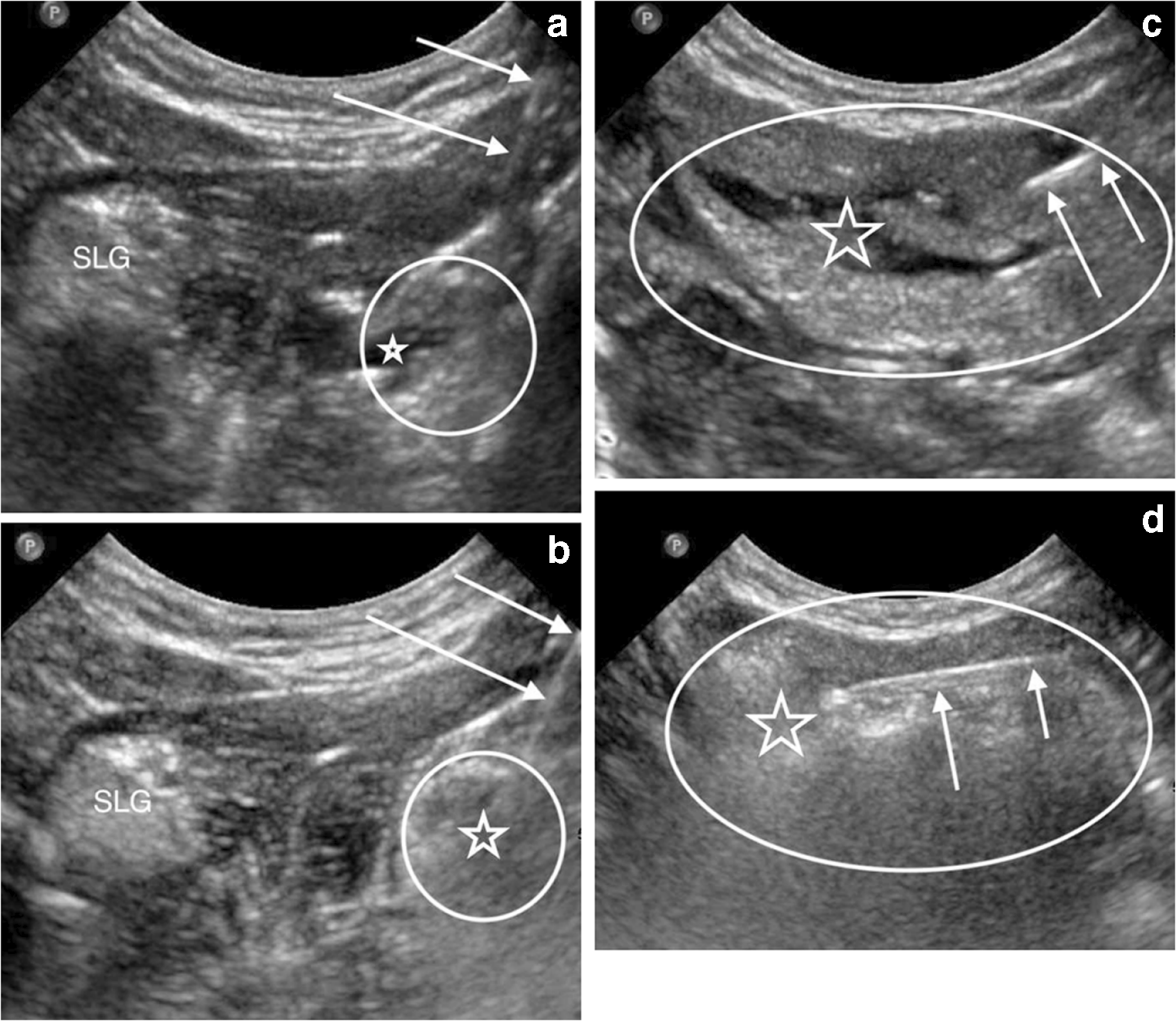
US images of the sublingual gland and submandibular gland during 3% sodium tetradecyl sulfate (STS) and 98% dehydrated ethanol (EtOH) injections in a 3-year-old girl. **a** Coronal US image from beneath the chin shows a needle (*arrows*) puncturing the right sublingual gland (*circle* outlines approximate margins of the gland) and injecting STS (*star*), which appears hypoechoic within the gland. The left sublingual gland (SLG) is labeled for comparison. **b** Coronal US image shows the needle (*arrows*) again puncturing the right sublingual gland (*circle* outlines approximate margins of the gland) and injecting EtOH (*star*), which causes immediate echogenic destruction of the gland. **c** US longitudinal to the submandibular gland (*oval* outlines the approximate margins of the gland) shows the needle (*arrows*) injecting STS (*star*), which causes the hypoechoic fissuring of the gland. **d** US longitudinal to the outlined (*oval*) submandibular shows the needle (*arrows*) injecting EtOH (*star*), which causes immediate echogenic destruction of the gland

The submandibular and sublingual glands were ablated using a 21- to 25-gauge needle that was advanced percutaneously from under the chin into the glands under direct US visualization. Un-foamed STS was injected throughout each gland while visualizing liquid distribution with US (Fig. 2). After a brief delay, EtOH was delivered in a similar fashion (Fig. 2). Care was taken to puncture the gland capsule as few times as possible. Drug volumes were determined by subtracting the residual syringe volume from the known initial syringe volume (Table 1).

**Table 1:** STS and EtOH injected volumes

Gland	STS (mL)	EtOH (mL)
Parotid gland	0.5–2.5	1.5–5.0
Submandibular gland	1.5–5.0	1.0–8.0
Sublingual gland	0.5–1.5	0.5–1.5

*EtOH* 98% dehydrated ethanol, *STS* 3% sodium tetradecyl sulfate

Injection into each gland was continued until a characteristic imaging response (good distribution of medication throughout gland) was achieved. The goal was to inject sufficient medication to see medication-induced changes in gland echotexture throughout the majority of the target gland. No numerical measurements of gland size were made to guide calculation of injected volumes. The glands are variably elastic and porous with injection. This fact, together with the operator-dependent nature of a procedure such as sclerotherapy, contributes to the variability of injected volumes. No facial nerve monitoring was used during any procedure.

Post-procedural prophylactic antibiotics were given for 7 days. For most patients, this was amoxicillin-clavulanic acid; patients with allergies precluding this were given clindamycin. Patients recovered in the post-procedure recovery unit and were discharged upon meeting standard discharge criteria. When appropriate, caregivers were advised to manage post-procedure pain with nonsteriodal anti-inflammatory drugs. Steroids were never prescribed at the time of discharge. They were rarely prescribed later by the interventional radiologist and more often by some of the patients’ other physicians if they felt the swelling was excessive. The exact frequency of later steroid prescription was not recorded.

### Study design

This retrospective review received institutional review board (IRB) approval and the requirement for informed consent was waived. All patients treated with salivary gland ablation at our institution between June 2005 and June 2019 were identified and their charts were reviewed. Two patient cohorts were devised: a larger one evaluated technical success and safety and a subcohort evaluated clinical efficacy. Inclusion criteria for the safety cohort required that each patient’s procedures and peri-procedural details were recorded in the medical record. This safety cohort includes early patients treated only with EtOH and later patients treated with our final dual drug method of STS and EtOH. Inclusion criteria for the efficacy cohort required that patients were referred for salivary gland ablation to treat anterior sialorrhea since most patients at our institution are referred for this indication. It is possible that some of these patients drool both anteriorly and posteriorly. Other inclusion criteria were that the patients had no other salivary gland surgeries, were treated with our final dual-drug method and completed our follow-up questionnaire. A letter that discussed the study and gave families the option to decline further contact was sent before any phone calls. Families were then called a week following the letter to obtain post-treatment outcome measures. Regular clinical follow-up usually terminated several months after the last treatment either with clinic visits or phone follow-up. For the purpose of the efficacy cohort created for this paper, follow-up phone calls were made at the time of this study, which accounts for the wide range in follow-up times since the treatments.

We retrospectively recorded patient age, gender, neurological diagnosis, salivary gland ablation procedure details and complications. Medications, treatments and surgeries were recorded for each patient if used to manage sialorrhea. Complications were categorized using the Society of Interventional Radiology (SIR) adverse events classification system [19]. This classification system ranks post-procedural complications from A to F, with A representing recovery that requires no additional therapy and has no consequences, and F representing death. Classifications B–D represent increasing levels of required therapy but with no permanent sequelae, and E represents permanent adverse sequelae resulting from the procedure.

The Drooling Frequency and Drooling Severity (DFDS) scale is a validated subjective drooling scoring system used by otolaryngology and neurology. It was used to determine drooling levels pre- and post-salivary gland ablation. This scale combines a drooling frequency score ranging from 1 to 4 and a severity scale ranging from 1 to 5, with the minimum possible DFDS score as 2 and the maximum as 9 [20]. Pre-scores were determined from the chart or retrospectively during follow-up phone interviews. Post-scores were exclusively determined by follow-up questionnaire administered via phone interview. The questionnaire elicited DFDS, side effects and whether the caregiver would elect to go through the procedure again given their retrospective feelings about the procedure and treatment outcome.

### Data analysis

We identified the number of technically successful procedures among all procedures that the patient cohort received, defining technical success as cannulation of either Stensen duct upon attempt. All targeted submandibular and sublingual glands were successfully injected. We compared pre- and post-procedure DFDS scores using a Student’s *t*-test. We determined there to be clinical value from the treatment if the caregiver answered “yes” to the question of whether they would choose to have the procedure again. Since the level of burden of sialorrhea on the patient and caregiver is highly individual and subjective, we believe this was an appropriate measure of clinical value. We compared post-procedure DFDS scores between patients whose treatments had clinical value and those without using a Student’s *t*-test. Continuous data were represented as means. Categorical data were represented as medians.

## Results

### Safety

One hundred and nine patients underwent salivary gland ablation between June 2005 and June 2019. Ninety-nine patients met our inclusion criteria for the safety cohort. These 99 patients underwent a total of 170 salivary gland ablation procedures. Forty-nine patients received 1 treatment (49.5%), 34 received 2 treatments (34.3%), and 16 patients received >3 treatments (16.1%). Patients had a median of four glands treated. We defined technical success as the ability to successfully complete the intended procedure. Due to the technical simplicity of injecting the submandibular and sublingual glands, which was achieved in all patients, we report only on the technical success of parotid gland treatment. The parotid gland injection was considered a technical success if the Stensen duct of either gland could be accessed. There were five procedures in which catheterization of both Stensen ducts needed to be attempted before one gland was successfully injected. Six procedures were considered technical failures because neither Stensen duct could be successfully cannulated. Salivary gland ablation was therefore technically successful 94.2% of the time.

All families reported significant swelling following the procedure. Twelve of the 170 procedures resulted in SIR adverse events Classification B; 5 overnight admissions for observation, 3 temporary nerve palsies (2 facial nerve, 1 marginal mandibular nerve), 2 abscesses requiring drainage (both adjacent to the treated parotid gland), 1 case of cellulitis and 1 requirement for increased anti-seizure medication following the procedure. Reasons for the nerve injuries were not discernable from the images. Most likely, there was some extravasation from the gland temporarily injuring an adjacent branch of the facial nerve. Two procedures were Class C, one for hospital admission due to desaturation and one procedure in which the patient experienced an unexplained respiratory arrest during the salivary gland ablation procedure and while under general anesthesia. Five procedures were Class D, three for prolonged hospital admission due to respiratory compromise and two for decreased oral intake. Patients with respiratory distress after their procedures did not have any identifiable clinical features separating them from the other patients in the cohort, such as greater risks for posterior drooling and aspiration. There were no patient deaths. In summary, 88.8% of procedures were SIR adverse events Class A, 7.1% were Class B, 2.0% were Class C and 2.9% were Class D. No procedures were classified Class E or F.

### Efficacy

Twenty-seven patients (14 males and 13 females) from the 99 patients in the safety cohort met our inclusion criteria into the efficacy cohort. Of the 72 patients who were excluded from the efficacy cohort, 11 were deceased when follow-up phone calls were attempted and the majority of the rest could not be contacted for follow-up.

The age range of this group was 6 to 29 years with a mean of 18 years at the time of follow-up. Mean time to follow-up phone interview was 5.4 years ranging from 57 days to 11.6 years (standard deviation [SD] ±3.36 years). Twenty-four (88.9%) patients’ primary neurological diagnosis was static encephalopathy, 2 (7.4%) were diagnosed with a non-static encephalopathy and 1 (3.7%) had myotonic dystrophy. All patients had been prescribed anticholinergic medication with unsatisfactory results. Three (8.8%) had tried BoNT-A at other institutions without achieving clinical satisfaction. None had prior salivary gland surgery.

Sialorrhea diminished after salivary gland ablation with the median pre-ablation DFDS score of nine decreasing to seven post-ablation (*P*=0.000018). Sixteen caregivers (59.3%) stated that they would choose to undergo the procedure if given the choice again, our definition of the treatment’s clinical value. These procedures yielded lower post-treatment DFDS scores than procedures in which the caregivers would not choose the procedure again. Patients in the group deemed to have clinical value had a median DFDS score of six post-procedure, while those in the group deemed not to have clinical value had a median score of eight post-procedure (*P*=0.0005). While it was not detailed why caregivers would elect not to repeat the procedure, our outcome data indicates that DFDS improvement is related to caregiver satisfaction.

In general, there was an association between the number of glands treated and the outcome. Of patients who had five (*n*=9) or six glands (*n*=4) ablated, a higher percentage of caregivers (69.2%) found clinical value from the procedure. There was no relationship between time to follow-up and outcomes, suggesting lasting treatment effects. The frequency of families who said the procedure was of value for their child was similar among patients who suffered an adverse event (55.6%) versus those who didn’t (61.1%). No caregivers reported mouth dryness in the patient at follow-up.

## Discussion

Sialorrhea is a common problem for children with neurodevelopmental disorders, which impacts the daily life of both child and caregiver. Frequent suctioning, instruction to swallow, and frequent bib, clothing and bedding changes are required to manage sialorrhea and its consequences [5]. Sialorrhea has a significant social and emotional impact on these children by limiting interaction with peers and family. Children with cerebral palsy and normal cognitive ability are often underestimated in their mental capacity due to sialorrhea [6]. Sialorrhea may not be the most severe symptom for children with complex disorders but decreasing its impact can profoundly improve their quality of life [5].

Saliva is produced by six major glands: paired submandibular, sublingual and parotid glands. Healthy adults produce approximately 1–1.5 L of saliva daily. Without sensory or kinetic stimulation, saliva distribution is 20% from the parotid glands, 65% from the submandibular glands, 7–8% from the sublingual glands and ~10% from minor salivary glands. Upon physiological stimulation, the parotid glands produce >50% of saliva. Acinar cells in the glands produce saliva that is then expelled from the acinar network through a duct system [21]. This would suggest that children who do not take oral nutritional intake would have symptoms more related to saliva produced from the submandibular glands making these glands a more important target of treatment in this subpopulation of patients. At least in our population of patients, many of the children who don’t eat receive alternate near-constant oral stimulation from their hands or bruxism. The major source of their drooling is therefore impossible for us to reliably determine and we believe treating all of the glands has a better chance of success than targeting just the submandibular glands or parotid glands.

Children with sialorrhea are first managed conservatively with postural and behavioral changes. Nonambulatory children are reclined to prevent spillage, and children who can obey commands are trained to better manage oral contents. Behavioral intervention is time-intensive, often yielding only transient improvement [9]. Anticholinergic medications can be effective, but side effects commonly limit their use [10, 11]. Surgical intervention for sialorrhea includes rerouting the submandibular gland ducts, ligating the submandibular ducts or parotid ducts, excising the submandibular glands and sublingual glands, and laryngotracheal separation. Submandibular duct rerouting is contraindicated in children who aspirate. Four-duct ligation (parotid and submandibular ducts bilaterally) is less invasive and carries fewer risks, but the recurrence rate can be up to 69% [13]. Bilateral submandibular gland excision leaves facial scars and carries risks of permanent nerve damage, hemorrhage and oral dryness [13]. Laryngotracheal separation with permanent tracheostomy is the most rare and extreme surgical method of preventing posterior sialorrhea in the event of persistent aspiration. This surgery renders patients unable to phonate, which limits caregiver acceptance of this approach [12]. The response rate when combining the variety of surgical treatments for sialorrhea is reported to be 81.6% by caregiver subjective scales, and the complication rate can be up to 40% [22–24]. The measurement and reporting of surgical complications and adverse events are heterogeneous and provide little ground for comparison with the cohort presented in this paper.

Percutaneous BoNT-A injection is an effective intervention with response rates of 80–91% and a complication rate of approximately 3–4% [25, 26]. This method of treatment typically requires general anesthesia for a pediatric patient and the effect of the drug wanes 4–6 months post-treatment [2]. A pediatric patient receiving BoNT-A injections at 6-month intervals between ages 6–21 years would require 30 injections. More commonly, patients receive injections at a larger average interval (9.8 months) and far fewer times on average (range: 3–11) [27]. This suggests that patients are not achieving effective long-term control of sialorrhea with BoNT-A injections.

Given the limitations of current behavioral, medical and surgical treatments for sialorrhea, we posited that sclerotherapy of the salivary glands might be a treatment alternative. Scientific support for this contention includes EtOH salivary gland ablation of the submandibular glands in a rat model where decreased acinar density, decreased glandular tissue, and increased fibrous tissue and fat necrosis were seen in treated submandibular glands [17]. Similar histological results are seen following submandibular gland duct ligation in rats and cryoablation of the parotid gland in pigs [28, 29]. We extrapolated that successful salivary gland ablation is possible in children and that sclerotherapy-induced acinar destruction would lead to decreased sialorrhea.

In this study, we demonstrate that salivary gland ablation has a low complication rate. There were no mortalities or permanent complications. Including complications from all SIR classes, the complication rate for salivary gland ablation was approximately 12% compared to 4% reported for Botox injections [26]. It is worth noting that none of the complications in our safety cohort in this study was permanent. The greater complication risk with salivary gland ablation comes with the greater potential for long-term symptom relief. Until such time as there is a randomized trial comparing BoNT-A injections to salivary gland ablation, it is impossible to say with certainty which approach strikes the best balance of immediate efficacy, temporary and permanent complication, long-term symptom relief and cost of care.

The risk of nerve injury is a well-recognized complication of submandibular gland surgery but was rare in our patients at 1.76%. There were three transient nerve injuries in our safety cohort — one marginal mandibular nerve and two facial nerves. Retrograde injection of sclerosant into the Stensen duct and through the parotid acinar network is a new technique allowing the drug to substantially penetrate the gland while minimizing the risk of nerve injury. The parotid glands are the largest source of stimulated saliva, and even children who are not fed orally may kinetically stimulate the glands with fingers and other objects. Other treatments for sialorrhea typically avoid the parotid gland directly due to the risk of nerve injury, and this has previously been a shortcoming in sialorrhea management [30]. The low rate of nerve injury in our safety cohort is encouraging and refining this technique is important for the future management of sialorrhea.

Due to the inflammation induced by each sclerosant, significant facial swelling occurred in all patients in our safety cohort, and this led to respiratory compromise in four patients. Our retrospective data did not specifically evaluate pain associated with the treatment, which would be very hard to assess in many of the nonverbal patients. It can be stated that it is not our clinical routine for patients to be discharged with any pain-relieving medication nor is it routine for any admitted patients to receive any pain medication during recovery. Any narcotic use in this population, many with baseline impaired respiratory reserve, would require caution. In general, despite sometimes profound swelling, families do not report perceived pain or agitation from the procedure.

Interestingly, SIR adverse event classification was not correlated with clinical value in our efficacy cohort. Patients with complications classified as B–D were as likely to be satisfied with the procedure and recovery process when compared to those who did not suffer any adverse events (61.1% and 55.6%, respectively). These data indicate that the severity of the complications would not deter the patients from undergoing the procedure. While oral dryness is a side effect of other sialorrhea treatments, none of our caregivers reported oral dryness as a long-term side effect.

Our data demonstrate that salivary gland ablation is an effective intervention for sialorrhea. Patients who underwent salivary gland ablation had significantly reduced DFDS scores post-procedure as compared to pre-procedure. The mean time to a follow-up phone interview was 5.4 years post-procedure, indicating that salivary gland ablation elicits a durable decrease in sialorrhea. A greater number of glands injected was associated with a greater decrease in DFDS and increased caregiver satisfaction. We believe this relationship supports the claim that salivary gland ablation is effective. Importantly, approximately 60% of caregivers said they would elect to have the procedure done again when considering the recovery process and long-term benefit.

The limitations of this study are those inherent to retrospective studies and small sample sizes. Many patients in our cohort were lost to follow-up. Our primary outcome measure, the DFDS, is a subjective measurement that limits the strength of the conclusions that may be drawn about the procedure’s effectiveness. However, the impact of sialorrhea on a patient or caregiver’s life is also not objectively measurable, so we feel that a subjective outcome measure is appropriate. While all eligible patients were contacted, we only included those who answered our phone call; therefore, self-selection bias may confound our findings. Pre-scores that were collected retrospectively are also subject to recall bias.

Future directions for salivary gland ablation research include a detailed prospective comparison of patients undergoing salivary gland ablation to those who undergo surgical management of sialorrhea. Such a study would be hard to pursue given the different types of surgeries performed, but it would shed light on the relative success, risk and cost of these treatment options. Such a study would need to consider recovery times from the different procedures and the costs of multiple ablations versus surgeries. An additional avenue for study would be children with posterior sialorrhea, with future studies on this population examining hospitalizations, aspiration pneumonias and survival as outcome measures following salivary gland ablation.

## Conclusion

Salivary gland ablation is a potential treatment for sialorrhea in children that is minimally invasive with a low procedural complication rate. Salivary gland ablation significantly decreases DFDS scores post-procedure and the effect seems to be stable with long-term follow-up.

## References

[1] Blasco PA, Allaire JH (1992). Drooling in the developmentally disabled: management practices and recommendations. Dev Med Child Neurol.

[2] Reddihough D, Erasmus CE, Johnson H (2010). Botulinum toxin assessment, intervention and aftercare for paediatric and adult drooling: international consensus statement. Eur J Neurol.

[3] Reid SM, McCutcheon J, Reddihough DS, Johnson H (2012). Prevalence and predictors of drooling in 7- to 14- year-old children with cerebral palsy: a population study. Dev Med Child Neurol.

[4] Erasmus CE, Van Hulst K, Rotteveel LJ (2009). Drooling in cerebral palsy: hypersalivation or dysfunctional oral motor control?. Dev Med Child Neurol.

[5] Van der Burg JJ, Jongerius PH, Van Hulst K (2006). Drooling in children with cerebral palsy: effect of salivary flow reduction on daily life and care. Dev Med Child Neurol.

[6] van der Burg JJ, Jongerius PH, van Limbeek J (2006). Social interaction and self-esteem of children with cerebral palsy after treatment for severe drooling. Eur J Pediatr.

[7] Faria J, Harb J, Hilton A (2015). Salivary botulinum toxin injection may reduce aspiration pneumonia in neurologically impaired children. Int J Pediatr Otorhinolaryngol.

[8] Jongerius PH, van Hulst K, van den Hoogen FJ, Rotteveel JJ (2005). The treatment of posterior drooling by botulinum toxin in a child with cerebral palsy. J Pediatr Gastroenterol Nutr.

[9] Little SA, Kubba H, Hussain SS (2009). An evidence-based approach to the child who drools saliva. Clin Otolaryngol.

[10] Hockstein NG, Samadi DS, Gendron K, Handler SD (2004). Sialorrhea: a management challenge. Am Fam Physician.

[11] Reid SM, Westbury C, Guzys AT, Reddihough DS (2019) Anticholinergic medications for reducing drooling in children with developmental disability. Dev Med Child Neurol. 10.1111/dmcn.14350 10.1111/dmcn.14350 31495925

[12] Hafidh MA, Young O, Russell JD (2006). Intractable pulmonary aspiration in children: which operation?. Int J Pediatr Otorhinolaryngol.

[13] Lawrence R, Bateman N (2018). Surgical management of the drooling child. Curr Otorhinolaryngol Rep.

[14] Gurgacz S, Zamora L, Scott NA (2014). Percutaneous sclerotherapy for vascular malformations: a systematic review. Ann Vasc Surg.

[15] Wijnands TF, Gortjes AP, Gevers TJ (2017). Efficacy and safety of aspiration sclerotherapy of simple hepatic cysts: a systematic review. AJR Am J Roentgenol.

[16] Gong X, Zhou Q, Chen S (2017). Efficacy and safety of ultrasound-guided percutaneous polidocanol sclerotherapy in benign predominantly cystic thyroid nodules: a prospective study. Curr Med Res Opin.

[17] Burch E, Lubeley L, Murakami J (2017). Percutaneous salivary gland ablation using ethanol in a rat model. J Oral Maxillofac Res.

[18] Brannan ZJ, Lubeley LJ, Sutphen SA, Murakami JW (2019). Percutaneous treatment of ranulas: ultrasound- guided drainage with salivary gland chemical ablation. Pediatr Radiol.

[19] Sacks D, McClenny TE, Cardella JF, Lewis CA (2003). Society of Interventional Radiology clinical practice guidelines. J Vasc Interv Radiol.

[20] Thomas-Stonell N, Greenberg J (1988). Three treatment approaches and clinical factors in the reduction of drooling. Dysphagia.

[21] Humphrey SP, Williamson RT (2001). A review of saliva: Normal composition, flow, and function. J Prosthet Dent.

[22] Reed J, Mans CK, Breitzke SE (2009). Surgical management of drooling: a meta-analysis. Arch Otolaryngol Head Neck Surg.

[23] Becmeur F, Schneider A, Flaum V (2013). Which surgery for drooling in patients with cerebral palsy?. J Pediatr Surg.

[24] Bekkers S, Delsing CP, Kok SE (2019). Randomized controlled trial comparing botulinium vs surgery for drooling in neurodisablilties. Neurology.

[25] Taib BG, Williams SP, Sood S (2019). Treatment of sialorrhoea with repeated ultrasound-guided injections of botulinum toxin A into the parotid and submandibular glands. Br J Oral Maxillofac Surg.

[26] Vashishta R, Nguyen SA, White DR, Gillespie MB (2013). Botulinum toxin for the treatment of sialorrhea: a meta-analysis. Otolaryngol Head Neck Surg.

[27] Sillanpää S, Sipilä M, Numminen J, Rautiainen M (2015). The experience of treating drooling with repeated botulinum toxin injections. ORL J Otorhinolaryngol Relat Spec.

[28] Standish SM, Shafer WG (1957). Serial histologic effects of rat submaxillary and sublingual salivary gland duct and blood vessel ligation. J Dent Res.

[29] Buethe JY, Abboud S, Brock K (2016). Percutaneous CT-guided cryoablation of the salivary glands in a porcine model. J Vasc Interv Radiol.

[30] Woo SH (2016). Endoscope-assisted transoral accessory parotid mass excision. Head Neck J.

